# Upcoming treatments for morphea

**DOI:** 10.1002/iid3.475

**Published:** 2021-07-17

**Authors:** Dan Wenzel, Nazgol‐Sadat Haddadi, Khashayar Afshari, Jillian M. Richmond, Mehdi Rashighi

**Affiliations:** ^1^ Department of Dermatology University of Massachusetts Medical School Worcester Massachusetts USA

**Keywords:** clinical trial, localized scleroderma, morphea, treatment

## Abstract

Morphea (localized scleroderma) is a rare autoimmune connective tissue disease with variable clinical presentations, with an annual incidence of 0.4–2.7 cases per 100,000. Morphea occurs most frequently in children aged 2–14 years, and the disease exhibits a female predominance. Insights into morphea pathogenesis are often extrapolated from studies of systemic sclerosis due to their similar skin histopathologic features; however, clinically they are two distinct diseases as evidenced by different demographics, clinical features, disease course and prognosis. An interplay between genetic factors, epigenetic modifications, immune and vascular dysfunction, along with environmental hits are considered as the main contributors to morphea pathogenesis. In this review, we describe potential new therapies for morphea based on both preclinical evidence and ongoing clinical trials. We focus on different classes of therapeutics, including antifibrotic, anti‐inflammatory, cellular and gene therapy, and antisenolytic approaches, and how these target different aspects of disease pathogenesis.

## Graphical abstract

Morphea is a rare connective tissue disease with variable clinical presentations, all of which are characterized by inflammatory fibrosis. No cure for morphea exists, but advances in our understanding of the mediators and cellular pathways underlying fibrosis have revealed potential therapeutic targets. Here, we review the upcoming treatments for morphea and other related inflammatory fibrosing disorders, focusing on anti‐inflammatory, antifibrotic, and senolytics approaches.

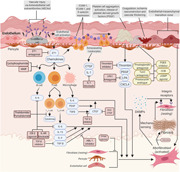

## 1 INTRODUCTION

Morphea (localized scleroderma) is a rare autoimmune connective tissue disease with variable clinical presentations, with an annual incidence of 0.4–2.7 cases per 100,000. Morphea occurs most frequently in children aged 2–14 years, and the disease exhibits a female predominance.^1^ Morphea is typically found in patches and bands with thickened skin on the head, extremities, and trunk. Although morphea is considered a skin‐limited disease, depending on the subtype and the affected anatomical location, it can cause significant disfigurement (hyperpigmentation and skin atrophy) and physical impairment (joint contracture). In addition, patients with morphea on head are at risk of ocular and neurological complications. There are currently efforts underway to update and improve the disease classification but morphea is generally classified into five groups of plaque, generalized, bullous, linear, and deep. The theories on its pathogenesis are often extrapolated from studies of systemic sclerosis (SSc) due to their similar skin histopathologic features; however, clinically they are two distinct diseases as evidenced by different demographics, clinical features, disease course, and prognosis.^2^, ^3^, ^4^, ^5^, ^6^, ^7^, ^8^, ^9^, ^10^, ^11^ An interplay between genetic factors, epigenetic modifications, immune and vascular dysfunction, along with environmental hits are considered as the main contributors to morphea pathogenesis.^12^

No cure for morphea exists, but advances in our understanding of the mediators and cellular pathways underlying fibrosis have revealed potential therapeutic targets to prevent permanent damage. Current treatment recommendations for morphea are limited, with a combination of systemic corticosteroids and methotrexate as the most common treatment option.^5^, ^9^, ^10^ However, these are not disease‐specific treatments and their long‐term use is associated with many side effects.^13^, ^14^ Thus, there are unmet needs in both understanding morphea pathogenesis and identifying targeted therapies. Here, we review the new emerging treatments for morphea (Table 1) focusing on potential novel treatments based on preclinical studies or early‐phase clinical trials for both morphea and cutaneous manifestations of SSc (Table 2). We discuss the rationale of investigations, including potential mechanisms of action and efficacy for specific clinical subtypes of morphea.

**Table 1:** Morphea treatments

Study title/number	Study design	Study population	Interventions	Results/outcomes	Mechanism of drug
*Antifibrotic targets*					
Efficacy and safety of imatinib in scleroderma (SCLEROGLIVEC) (NCT00479934)	Randomized, double blinded, placebo controlled trial, phase 2, 24‐week	28 participants age ≥18 with either systemic or cutaneous scleroderma, severe cutaneous involvement (m‐Rodnan score >20/51)	Imatinib 400 mg/day versus placebo, for 6 months	Completed, awaiting results; **Primary outcome measures** Percent variation of modified Rodnan score between inclusion and 6‐month visits **Secondary outcome measures** • Percent variation of modified Rodnan score at 1, 3, and 12 months • Skin thickness at inclusion and 6 month using skin biopsies • DLQI and HAQ assessed at 1, 3, 6, and 12 months • Tolerance of treatment • Effect of treatment on noncutaneous symptoms	Bcr‐abl tyrosine kinase inhibition
Study to assess sarilumab in halting progression of morphea (NCT03679845)	Open label, single group assigned trial, phase 1/2, 24‐week	20 participants age ≥18 with plaque‐type morphea, at least one active morphea lesion (0.5–10 cm), mLoSSI ≥5, ≤50% of body surface area being affected	Sarilumab 200mg, injected IV every 2 weeks over 24 weeks	Actively recruiting; **Primary outcome measures** Efficacy of sarilumab **Secondary outcome measures** Physician global assessment of activity (PGA‐A)	IL‐6 receptor antibody
*Anti‐inflammatory targets*					
Imiquimod in children with plaque morphea (NCT00147771)	Nonrandomized, single group open‐label trial, Phase 3, 48‐week	10 participants between the ages of 6 and 18 with morphea plaques	Imiquimod 5% cream, applied 3–5 times a week for 24 weeks	Completed, awaiting results; **Primary outcome measures** Percent improvement in thickness of skin **Secondary outcome measures** Frequency of side‐effects	Toll‐like receptor 7 agonist
Pilot study evaluating the efficacy of a topical PDE4 Inhibitor for morphea (NCT03351114)	Open label, single arm, trial, phase 2, 20‐week	20 participants ≥18 years with clinical diagnosis of morphea involving <20% total body surface area	Crisaborole 2% ointment, applied twice per day for 12 weeks	Actively recruiting; **Primary outcome measures** Change in dermal thickness of sentinel plaque (using 4mm punch biopsy) **Secondary outcome measures** • Change in dyspigmentation, induration, erythema, and telangiectasias_DIET score and dermal thickness (using ultrasonography) of sentinel plaque • Change in Localized Scleroderma Cutaneous Assessment Tool—LOSCAT score, and health‐related quality of life (Skindex‐29)	PDE4 inhibitor
Clinical trial to evaluate efficacy and safety of dupilumab in localized scleroderma (NCT04200755)	Randomized, multicenter, double‐blind, placebo controlled, parallel group trial, phase 2, 24‐weeks	45 participants ≥18 years with either plaque type morphea or generalized localized scleroderma affecting at least three anatomic sites Must have at least one lesion with lilac ring (active phase of disease) and activity of LS within the last 12 month (as defined by progression of size or new plaque	Dupixent 300 mg (30 patients) vs. placebo (15 patients), initiated with two simultaneous SC injections followed by SC injection every 14 days for 24 weeks	Not yet recruiting; **Primary outcome measures** Change in Localized Scleroderma Cutaneous Assessment Tool—LOSCAT score of target lesion (from baseline to end of treatment visit, 24 weeks) **Secondary outcome measures** From baseline to follow‐up visit, 48 weeks: • Change in mLoSSI (Localized Scleroderma Skin Activity Index) of all lesions • Change in LoSDI (Localized Scleroderma Skin Damage Index) of all lesions • Number of lesions • Change in DermatoLogy Quality of life Index (DLQI) • AEs • Others: Physical examination, Body weight, blood pressure, pulse rate, body temperature, hemoglobin, hematocrit, platelet count and WBC with differential, blood enzymes, clinical chemistry, ANA antibody levels, serum cytokine levels From baseline to end of treatment visit, 24 weeks: • RNAseq data • RT‐qPCR data	IL‐4/IL‐13 inhibitor
Molecular effects of topical calcipotriene on morphea (NCT02411643)	Open label, single group assigned trial, early phase 1, 12‐week	2 participants age ≥18 with clinically diagnosed or biopsy proven plaque‐type, guttate, linear, segmental, and generalized morphea that are receiving calcipotriene 0/005% ointment	Calcipotriene 0.005% ointment, applied twice per day for 3 months	Terminated; **Primary outcome measures** Change of gene expression from skin biopsy **Secondary outcome measures** • Quality of life • Modified localized scleroderma skin score • Change of appearance of skin biopsy	↑ TSLP by keratinocytes, repress Th1/Th17 inflammation
Comparative effectiveness trial in the treatment of pediatric plaque morphea (NCT02680717)	Nonrandom, single group assigned trial, phase 1, 8‐week	0 participants enrolled—study terminated	Calcipotriene 0.005% ointment or tacrolimus 0.1% ointment, applied twice per day or clobetasol 0.05% ointment, applied twice daily (alternating weeks)	Terminated; **Primary outcome measures** Visual analog scale at 2 and 4 months	Calcipotriene: synthetic vitamin D3 analog Clobetasol: a corticosteroid Tacrolimus: calcineurin inhibitor
A protocol based treatment for debilitating fibrosing skin disorders with (anti‐CD 20), rituximab, evaluating safety and efficacy (NCT00936546)	Nonrandom, open label, single group assigned trial, phase 2, 5‐year	10 participants age ≥18 with a disabling fibrosing skin disorder not fulfilling the ACR criteria for diffuse SSc and inadequate response to methotrexate	Rituximab 1000 mg (Mabthera), injected IV at baseline and at month 6	Unknown, awaiting results; **Primary outcome measures** Safety at baseline, month 3, 6, 12, 15, 18, 24, 36, 48, and 60. **Secondary outcome measures** Efficacy at baseline, month 3, 6, 12, 15, 18, 24, 36, 48, and 60.	Anti‐CD20
Imiquimod 5% cream in plaque‐type morphea: a pilot, prospective open‐label study (NCT00230373)	Non‐randomized, open label, single group assigned trial, phase 3, 1‐year	20 participants age ≥6 with plaque‐type morphea	One plaque treated with imiquimod 5% cream, and another lesion with placebo (vehicle cream) for 9 months	Withdrawn; **Primary outcome measures** Improvement of skin induration evaluated by ultrasound **Secondary outcome measures** Adverse outcomes	Toll‐like receptor 7 agonist
*Miscellaneous mechanism*					
A study of FCX‐013 plus veledimex for the treatment of moderate to severe localized scleroderma (NCT03740724)	Open label, single group assigned, trial, phase 1/2, 20‐month	Patients age ≥18 and with clinical diagnosis of localized scleroderma being treated with a stable course of systemic immunosuppressive therapy	FCX‐013 injected intradermally 1–2 times (12 weeks apart) + veledimex initiated on the day of injection and continued for 2 weeks	Actively recruiting; **Primary outcome measures** Safety **Secondary outcome measures** • Skin thickness and fibrosis measured by: ∘∘ histology compared to day 0 ∘∘ (week 12 and 25) ∘∘ ultrasound/durometry compared to day 0 (week 4, 12, 17, and 25) ∘∘ MRI compared to day 0 (week 4, 12, 17, and 25) • Skin thickness component of mLoSSI of sentinel lesion • Skin thickness by mRSS compared to day 0 (week 4, 8, 12, 16, 17, 21, 25, and 29)	FCX‐013 is genetically modified fibroblast that secretes MMP1 in response to Veledimex
Efficacy and safety of PLACENTEX ® IM in patients with scleroderma diseases (NCT03388255)	Open label, single group assigned, trial, phase 4, 24‐week	25 participants age ≥18 with localized scleroderma diseases during inactive stage with fibrotic and atrophic cutaneous lesions confirmed histologically	Polydeoxyribonucleotide (PLACENTEX ®) 5.625 mg/3 ml, injected i.m. per day for 12 weeks	Actively recruiting; **Primary outcome measures** Localized Scleroderma Cutaneous Assessment Tool—LOSCAT score **Secondary outcome measures** • Change in tele‐thermographic profile (24 weeks) • Change in ultrasound profile of target cutaneous lesion (24 weeks) • Measurement of histology improvement (12 weeks) • DLQI (24 weeks)	?
*Phototherapy*					
Treatment study comparing UVA‐1 phototherapy versus placebo treatment for morphea (NCT01799174)	Randomized, triple blinded, placebo controlled, parallel assignment trial, 3‐year	14 participants age ≥6 with at least one active morphea lesion (linear, plaque, generalized, or mixed subtypes)	UVA‐1 (70 J/cm^2^) versus placebo (0 J/cm^2^), three times per week for 10 weeks	Completed, awaiting results; **Primary outcome measures** Change in LoSSI from baseline (from baseline vs. after 30 treatments) **Secondary outcome measures** • Physician's global assessment of disease activity (PGA‐A) over 3 years • Gene expression profiling over 3 years	UV mediatedimmunosuppression
Ultraviolet B (UVB) light therapy in the treatment of skin conditions with altered dermal matrix (NCT00129428)	Open label, single group assigned trial, phase 1/2, 16‐week	33 participants aged 10 or older with clinical diagnosis of scleroderma	UVB (290–320 nm at up to 320 mJ/cm^2^), at maximum five times per week for 16 weeks	Completed, awaiting results; **Primary outcome measures** Improvement in appearance of lesions (week 1, 2, 4, and then at monthly intervals until end of study) **Secondary outcome measures** • Assays to be performed on biopsy specimens • Photographs taken at baseline and end of study	UV mediatedimmunosuppression
Treatment study comparing medium or high dose Uva‐1 treatment 3×/week versus fluocinonide 0.05% cream in the treatment of morphea (NCT00812188)	Randomized, single blinded (investigator), parallel assigned trial, 5‐year	24 participants aged 18 or older with symmetric limited morphea	Fluocinonide 0.05% cream twice per day to one plaque for 12 weeks and, UVA‐1 medium dose (60 J/cm^2^) or high dose (120 J/cm^2^), 3×/week for 12 weeks to another plaque	Completed, awaiting results; **Primary outcome measures** Efficacy of UVA‐1 treatment versus topical steroid over 5 years	UV mediatedimmunosuppression
UVA‐1 light for scleroderma and similar conditions (NCT00476697)	Open label, single group assigned trial, 28‐week	23 participants aged ≥10 years with clinical diagnosis of keloid, scleroderma, acne keloidalis nuchae, old burn scars, granuloma annulare or other related conditions	UVA1 irradiation 5×/week for up to 16 weeks with dose increasing up to 130 J/cm2	Terminated; **Primary outcome measures** Plaque thickness, increase in mobility, and plaque hardness (at 16 weeks) **Secondary outcome measures** Analysis of collagen levels and MMP induction (at 16 weeks)	UV mediatedimmunosuppression
Fractional carbon dioxide laser versus UVA1 phototherapy for treatment of localized scleroderma: a clinical & immunohistochemical comparative study (NCT02002897)	Randomized, open label, parallel assigned, trial, 12‐week	20 participants at any age with plaque, linear, or atrophic morphea	Fractional carbon dioxide laser 10,600 nm, 1×/month for 3 months versus UVA1 (340–400 nm) 3×/week for 8 weeks	Unknown status; **Primary outcome measures** Efficacy of fractional carbon dioxide laser (over 12 weeks) **Secondary outcome measures** • Degree of improvement of cases of localized scleroderma (over 12 weeks) • Complications (over 4 months)	UV mediatedimmunosuppression

*Note:* A comprehensive table of all current interventional clinical trials for morphea listed on clinicaltrials.gov. The table is broken down based on the mechanism of intervention being used as well as the status of the trial (e.g., *completed, active, terminated*, etc.).

**Table 2:** Cutaneous SSc treatments

Study title/number	Study design	Study population	Interventions	Results/outcomes	Mechanism of drug
*Antifibrotic targets*					
High dose cyclophosphamide for treatment of scleroderma NCT00501995	Open label, single group assigned clinical trial, 24‐month	Six participants with mean age of 39 years and female to male ratio of 0.33 (33.3%) All participants met criteria for diffuse cutaneous scleroderma with clinical evidence of active disease that is deforming and threatens their capacity to function normally in society	Cyclophosphamide (50 mg/kg) Intravenous for 4 consecutive days	Completed, with results; *(6 patients started treatment, one patient died during the early phase.)* **Primary outcome measures** Improvement in mRSS from baseline (measured at 0, 1, 3, 6, 12, and 24 months; >25% is considered significant): 46.75% improvement from baseline **Secondary outcome measures** Change in HAQ‐DI (The Health Assessment Questionnaire‐Disability Index (HAQ‐DI); pre and post study percentages were compared: 79% improvement from baseline Change in physician global assessment (The physician global assessment (PGA) which is a visual analogue scale from 0 to 100 on which the physician rates the patient's disease severity based on their observations. 71% improvement from baseline	DNA alkylating agent
Comparison of therapeutic regimens for scleroderma interstitial lung disease (The Scleroderma Lung Study II) (SLSII) NCT00883129	Quadruple blinded, parallel assigned, placebo controlled phase II RCT, 24‐month	142 participants with mean age of 52.3 (*SD*, 9.7), female to male ratio of 105 to 37 (73.9% female), mean duration of scleroderma of 2.6 years (1.8) and mean mRSS of 14.7 (10.5). 83 (58.5%) of participants had diffuse cutaneous scleroderma and 59 (41.5%) had limited cutaneous scleroderma	Mycophenolate arm: 24‐months of oral MMF, up to max dose of 1.5 g twice daily. Experimental arm (Exp.): 12‐months of oral cyclophosphamide, up to max dose of 2 mg/kg/day plus 12 months of placebo during 2nd year of treatment	Completed, with results (significant result defined as *p* ≤ .05; **Primary outcome measures** FVC (measured at 3, 6, 12, 15, 18, 21 and 24 months)] 1. A modified ITT inferential joint model: ***p*** = **0.24** 2. Same model as first ITT model but for each treatment arm independently: ***p*** ≤ **.05** 3. Based on the absolute difference between the value of FVC%: ***p*** = **.55** **Secondary outcome measures** Total lung capacity (measured at baseline, 6, 12, 18, and 24 months): ***p*** **≥ .05** DLCO • MMF vs Exp.: ***p*** ≤** .001** • MMF group: ***p*** ≥ **.05** Fibrosis score, as measured with high res. CT scan (analysis was carried out in the subset of subjects that had measurable HRCT scans at both study entry and 24 months) • MMF group: ***p*** ≥ **.05** Transitional Dyspnea Index Score (measured at 6, 12, 18, and 24 months) • MMF group: ***p*** ≥** .05** • MMF versus Exp.: ***p*** ≤ **.05** Skin involvement, as measured by mRSS (measured at baseline, 3, 6, 9, 12, 15, 18, 21, and 24 months) • MMF versus Exp.: ***p*** ≥** .05** • MMF versus Exp. (each treatment arm independently analyzed via same inferential joint model): ***p*** ≤ **.05** • MMF versus Exp (done for each individual subject: ***p*** ≥ **.05** Toxicity, as measured by AEs, SAEs, and death (MMF vs. Exp): ***p*** < **.05** Tolerability, as Assessed by the time to withdrawal from the study drug or meeting protocol‐defined criteria for treatment failure (MMF vs. Exp): ***p*** = **.019**	MMF = depletes guanosine nucleotides preferentially in T and B lymphocytes and inhibits their proliferation Cyclophosphamide = DNA alkylating agent
Safety, tolerability, efficacy, and pharmacokinetics of JBT‐101 in systemic sclerosis NCT02465437	Double blinded, parallel assigned, placebo controlled RCT, 16‐week	42 participants Baseline mRSS = 24.5 (10.60) Baseline HAQ‐DI = 1.26 (0.809) Mean age = 47.9 (10.57) years Female:male = 3.2 (76.2%)	JBT‐101(Lenabasum) JBT‐101 5 or 20 mg q am and placebo q pm on Days 1–28, then JBT‐101 20 mg twice a day (bid) on Days 29–84. JBT‐101 20 mg or placebo bid on Days 1–84. JBT‐101 20 mg bid on Days 1–364. All treatments given orally	Active, not recruiting, results posted; **Primary outcome measures** Number of participants with treatment‐emergent AEs from baseline to day 113 (active dosing Days 1–84; plus 28 day follow‐up): Combined JBT‐101 group: *n* = 17 (63%) Placebo group: *n* = 9 (60%) Combined response index in diffuse cutaneous systemic sclerosis (CRISS) at day 85 and 113 (higher CRISS score indicates improvement); outcome is a continuous variable between 0.0 and 1.0; A cut‐off at 0.6 in the predicted probability of being improved has yielded the smallest misclassification error: Placebo (Day 85): 0.010 Placebo (Day 113) 0.00 JBT‐101 (Day 85): 0.275 JBT‐101 (Day 113): 0.330 **Secondary outcome measures** CRISS individual components (mRSS Total Score) change from baseline. mRSS consists of an evaluation of patient's skin thickness rated by clinical palpation using a 0–3 scale: 0 = normal skin; 1 = mild thickness; 2 = moderate thickness; 3 = severe thickness with inability to pinch the skin into a fold for each of 17 surface anatomic areas of the body. Total score is 0–51 JBT‐101 (Day 85): −3.8 (1.1) JBT‐101 (Day 113: −4.7 (1.1) Placebo (Day 85): −2.6 (1.5) Placebo (Day 113): −2.0 (1.5) CRISS individual component (physician global assessment score) change from baseline (0–10 scale; higher number = worse symptomatology) JBT‐101 (Day 85): −0.9 (0.3) JBT‐101 (Day 113): −0.9 (0.3) Placebo (Day 85): −0.5 (0.4) Placebo (Day 113): −0.7 (0.3) CRISS Individual Component (HAQ‐DI Score) Change From Baseline (higher score = worse symptomatology) JBT‐101 (Day 85): −0.22 (0.07) JBT‐101 (Day 113): −0.14 (0.07) Placebo (Day 85): 0.11 (0.10) Placebo (Day 113): 0.11 (0.09)	Cannabinoid receptor type 2 agonist
Evaluation of tofacitinib in early diffuse cutaneous systemic sclerosis (dcSSc) (TOFA‐SSc) NCT03274076	Double‐blind, phase I/II, placebo controlled RCT, 24‐week	15 participants with mean baseline mRSS of 23.3 (8.4). Female:male ratio of 10:5 (66.7% female). Mean age of 50.8 years	5 mg tofacitinib twice a day for 24 weeks versus Placebo	Completed, results posted; **Primary outcome measures** Number of participants who experience grade 3 or higher AEs that occur at or before week 24: 0 AEs in either group **Secondary outcome measures** Number of grade 3 (severe) or higher AEs that occur throughout the study: 0 in either group at week 12 0 in either group at week 24 2 in tofacitinib group at week 36; 0 in placebo group 1 in tofacitinib group at week 48; 1 in placebo group Change in mRSS from baseline to week 12, 24, 36, and 48 *p* = .1978 (not significant between groups) Provisional ACR Combined Response Index (CRISS) Systemic Sclerosis at week 12, 24 and 48 *p* = .4535 (not significant between groups)	JAK‐inhibitor
Trial to evaluate efficacy and safety of lenabasum in diffuse cutaneous systemic sclerosis (RESOLVE‐1) NCT03398837	Double‐blind, phase 3, placebo controlled RCT, 52‐week	365 participants aged 18 years and older with ddSSc (involvement of upper arms, upper legs, or trunk) and <6 years duration since first non‐Raynaud's symptom.	Lenabasum (5 mg given twice per day and 20 mg twice per day) orally versus placebo	Active, not recruiting (without results); **Primary outcome measures** Efficacy of lenabasum compared to placebo (via ACR Combined Response Index score through study completion, up to 1 year) **Secondary outcome measures** Change in mRSS (from baseline up to 1 year) Change in HAQ‐disability index (from baseline up to 1 year) Change in FVC (from baseline up to 1 year)	Cannabinoid‐2 agonist
Effectiveness and safety of lidocaine for scleroderma NCT00740285	Double blinded, parallel assigned trial, phase2/3, 6‐month	26 participants aged 18 to 60 years old who have had diffuse or limited sScleroderma for <5 years	20 ml lidocaine 2% + 0.9% 500 ml IV for first 5 days; 30 ml lidocaine 2% + 0.9% 500 ml IV for next 5 days	Completed, awaiting results; **Primary outcome measures** Skin thickening evaluated by skin score (before, immediately after intervention and 6‐months later) **Secondary outcome measures (evaluated before, immediately after, and 6‐months after intervention)** Safety (adverse effects) Quality of life evaluated by HAQ Pressure at lower esophagus evaluated by esophagus manometry Vessel alterations evaluated by fingernail capillaroscopy Subjective evaluation by patients	Inhibiting voltage‐gated sodium channel, inhibits activity of prolyl hydroxylase
Scleroderma lung study III—Combining pirfenidone with mycophenolate (SLSIII) NCT03221257	Double blinded, parallel assigned, placebo‐controlled, phase II RCT, 18‐months	150 participants age 18 years and older, with scleroderma as determined by the 2013 ACR/EULAR classification criteria, grade ≥2 on Magnitude of Task component of the Mahler Modified Dyspnea Index, FVC ≤ 85% at screening, onset of first non‐Raynaud manifestation of SSc within the prior 84 months, and presence of any ground‐glass opacification (GGO) on thoracic HRCT	PFD + Mycophenolate (MMF): PFD titrated up to 801 mg taken orally three times daily, (three‐step titration occurring at 2 week intervals) plus MMF 1500 mg orally twice daily (four‐step titration occurring at monthly intervals). versus Placebo (inactive capsule, resembles PFD) plus MMF 1500 mg twice daily (four‐step titration occurring at monthly intervals)	Actively recruiting; **Primary outcome measures** Percent predicted FVC (from baseline to 18 months, measured every 3 months) **Secondary outcome measures** Percent predicted single‐breath DLCO for carbon monoxide (from baseline to 18 months, measured every 3 months) mRSS (from baseline to 18 months, measured every 3 months) Mahler Modified Transitional dyspnea Index (TDI) (from baseline to 18 months, measured every 3 months) Health Assessment questionnaire modified for scleroderma (SHAQ) (from baseline to 18 months, measured every 6 months) St. George's Respiratory questionnaire (from baseline to 18 months, measured every 3 months) Scleroderma‐related interstitial lung disease measured via high resolution computerized tomography (HRCT) (from baseline to 18 months) Total lung capacity (from baseline to 18 months) The time (in months) required for each treatment arm to achieve a 3.0% or greater improvement from baseline in the FVC‐% over the 18‐month treatment period. A threshold analysis based on the % of subjects in each treatment arm achieving greater than a 5% improvement in FVC Time to withdrawal from the study or treatment failure (from baseline to 18 months) Number of participants with treatment‐related AEs as assessed by system organ classification (from baseline to 18 months)	Pirfenidone = reduces fibroblast proliferation, and inhibits TGF‐β stimulated collagen production MMF = depletes guanosine nucleotides preferentially in T and B lymphocytes and inhibits their proliferation
Safety and suitability of dabigatran to inhibit thrombin in scleroderma NCT02426229	Single group assigned, open label treatment clinical trial, 6‐month	15 participants aged 18–70 years old who fulfill the ACR/EULAR criteria for SSc with diagnosis for <7 years. Patients may have limited (cutaneous thickening distal, but not proximal to elbows and knees, with or without facial involvement) or diffuse (cutaneous thickening proximal to elbows and knees, often involving the chest or abdomen) cutaneous SSc, or systemic sclerosis sine scleroderma	Dabigatran etexilate 75 mg BID orally for 6 months	Completed, awaiting results; **Primary outcome measures** Safety of dabigatran patients with scleroderma interstitial lung disease. (complete blood counts, comprehensive metabolic profile, and coagulation studies). (time frame: up to 6 months) **Secondary outcome measures** Preliminary estimate of efficacy of dabigatran in scleroderma (skin score and dermal fibroblast biology to obtain preliminary estimates of the effectiveness of dabigatran as a potential disease modifying drug for patients with SSc‐ILD) (time frame: up to 6 months)	Competitive reversible nonpeptide antagonist of thrombin
Proof‐of‐concept trial of IVA337 in diffuse cutaneous systemic sclerosis (FASST) NCT02503644	Double blinded, placebo controlled RCT, 48‐week	145 participants aged 18 to 75 years old with Systemic sclerosis according to ACR/EULAR 2103 criteria^169^ and Diffuse cutaneous SSc subset according to LeRoy's criteria who have had diagnosis made within the past 3 years as defined by the first non‐Raynaud's symptom.	IVA337 (400 or 600 mg) BID orally versus placebo	Completed, awaiting results; **Primary outcome measures** Measurement of skin thickness by the mRSS (time frame: 48 weeks) **Secondary outcome measures** Response rates based on MRSS improvement (*Initial definition: improvers are defined by a reduction* ≥ *5 points and* ≥ *25% of MRSS; Additional definition: improvers are defined by a reduction)* (Time frame: 12, 24, 32, 48 weeks) Overall progression of the disease: defined as absence of rescue therapy and absence of severe organ involvement (time frame: 28, 32,40, and 48 weeks) Scleroderma Health Assessment Questionnaire (SHAQ) (time frame: 24 and 48 weeks) Patient‐reported health status assessed by PROMIS29 (time frame: 24 and 48 weeks) Physical and mental health assessed by SF36 (time frame: 24 and 48 weeks) *Please reference study for more secondary outcome measures*	PPAR activator
A study of the efficacy and safety of tocilizumab in participants with systemic sclerosis (SSc) NCT02453256	Double blinded, placebo controlled RCT, 48‐week	212 participants (105 to tocilizumab group (TCZ), 107 to placebo) with mean age of 48.2 years and female to male ratio of 4.38 (81.4%). Participants must have diagnosis of SSc according to ACR and EULAR criteria and meet criteria for active disease with total disease duration of ≤60 months	Tocilizumab 162 mg subcutaneous once weekly for 48 weeks versus placebo.	Completed, with results; **Primary outcome measures** Change in mRSS during double‐blind period between TCZ group and placebo *p* = .098 **Secondary outcome measures** Percentage of participants with ≥20%, ≥40%, or ≥60% improvement in mRSS during double‐blind period *p* = .0007 (*this statistical analysis applies to participants with* ≥ *20% improvement in mRSS)* *p* = .5139 (*This statistical analysis applies to participants with* ≥ *40% improvement in mRSS)* *p* = *.3276* (*this statistical analysis applies to participants with* ≥ *60% improvement in mRSS)*	IL‐6 receptor antibody
IL1‐TRAP, rilonacept, in systemic sclerosis NCT01538719	Quadruple blinded, parallel assigned, placebo‐controlled RCT, 6‐week	24 participants with mean age of 50.8 years; female to male ratio of 9–10 (47.4% female) and mRSS > =15	2:1 randomization: 12 patients received Rionacept 320 mg subcutaneously (SQ) on day 0 and 160 mg SQ each week for 5 additional weeks 7 patients received placebo (saline) subcutaneously on day 0 and each week for 5 additional weeks	Completed; with results **Primary outcome measures** Change in 2‐gene biomarker expression in skin (measured at visit 3 (day 42) and visit 1 (day 0) and calculated using a previously validated equation); the two genes (THBS1 and MS4A4) are measured via nanostring. A high biomarker score is equivalent to a high skin score (higher severity): Rilonacept group: 0.42 (−2.97–3.82) Placebo: −2.67 (−5.92–0.57) **Secondary outcome measures** Change in mRSS (visit 3 (day 42)–visit 1 (day 0)) Rilonacept group: −0.4 (−5–9) Placebo: −0.6667 (−5–14)	IL‐1β inhibitor
Study of pomalidomide (CC‐4047) to evaluate safety, tolerability, pharmacokinetics, pharmacodynamics and effectiveness for patients with systemic sclerosis with interstitial lung disease NCT01559129	Double blind, placebo controlled, phase II RCT, 156‐week	23 participants, age 18–80 years with diagnosis of SSc and onset of first non‐Raynaud's manifestation within 7 years of screening	Pomalidomide 1 mg orally once daily for 52 weeks during treatment phase for up to 2‐years during open label extension phase versus placebo	Study was terminated: Based upon interim analysis data, the study did not meet its primary endpoints for subjects who had completed blinded treatment. The IDMC recommended the study be stopped due to lack of efficacy and the sponsor agreed with this recommendation	Pomalidomide = targets protein “cereblon”; various immunomodulatory actions along with inhibition of TNF‐ɑ and IL‐6
Proof of concept trial of gleevec (imatinib) in active diffuse scleroderma NCT01545427	Double blinded, parallel assigned RCT, 6‐month	10 participants aged 18 and older who meet preliminary criteria for scleroderma and have diffuse skin involvement. Disease must appear to be active as measured by worsening skin score and/or increased ESR	Imatinib mesylate 200 mg BID orally for 6 months versus placebo	Study terminated (frequent AEs occurred early in treatment with poor tolerability)	Bcr‐abl tyrosine kinase inhibitor
*Anti‐inflammatory targets*					
A Study of Subcutaneous Abatacept to Treat Diffuse Cutaneous Systemic Sclerosis (ASSET) NCT02161406	Placebo controlled, double blinded RCT, 12‐month	88 participants with diagnosis of SSc as defined by 2013 ACR/European Union League Against Rheumatism classification plus Diffuse Systemic Sclerosis (dcSSc) as defined by LeRoy and Medsger, and a disease duration of ≤36 months (time from first non‐Raynaud phenomenon manifestation) For those with disease duration of ≤18 months, mRSS units at screening visit must be ≥10 and ≤35. For those with disease duration >18–36 months, mRSS units at screening must be ≥15 and ≤45 plus one of the following: Increase ≥3 in mRSS units compared with the last visit within previous 1–6 months Involvement of one new body area with ≥2 mRSS units compared with the last visit within the previous 1–6 months Involvement of two new body areas with ≥1 mRSS units compared with the last visit within the previous 1–6 months Presence of 1 or more Tendon Friction Rub	Abatacept 125 mg injections versus placebo weekly for 52 weeks	Completed, with results (significant value defined as *p* ≤ .05 **Primary outcome measures** Proportion of patients with at least one AE or SAE in 1 year Abatacept group: 79.5% Placebo group: 90.9% Change from baseline in mRSS to month 12 between groups *p* = .28 **Secondary outcome measures (from baseline to 12 months)** Patient global assessment for overall disease *p* = .73 Physician global assessment for overall disease ***p*** = **.03** Change in % Predicted FVC *p* = .11 Change in FVC (in ml) *p* = .19 Change in HAQ‐DI overall ***p*** = **.005** All other results are not statistically significant between treatment groups	Inhibits costimulatory signal of T‐cells by binding CD80/CD86
The effect of ethanol extract *Physalis angulata* Linn. in scleroderma patients with standard therapy NCT03141125	Double blinded, placebo controlled RCT, 3‐month	62 participants between age 15 and 60 who routine control in rheumatology outpatient clinics in Ciptomangunkusumo hospital Jakarta and Hasan Sadikin hospital Bandung RSHS who received standard therapy for scleroderma with a stable dose over the past 3 months. Participants must meet criteria of either limited or diffuse type scleroderma and have mRSS ≥ 5 and disease duration ≥1 year	*P. angulata* ethanol extract 3× 250 mg per day, given orally for 3 months versus placebo Patients also received standard therapy for scleroderma	Completed, awaiting results; **Primary outcome measures** Degree of skin fibrosis in scleroderma patients measured by mRSS over 3 months of intervention **Secondary outcome measures (over three months)** Level of P1NP serum (improvement = reduced P1NP) Value of ESR (improvement = reduced ESR) Level of BAFF (improvement = reduced BAFF) Level of sCD40L (improvement = reduced sDC40L)	Anti‐inflammatory—visit: https://www.ncbi.nlm.nih.gov/pubmed/18513903
A study of the safety and tolerability of MEDI‐551 in scleroderma NCT00946699	Double‐blinded, phase 1 RCT, 12‐week	50 participants aged 18 years or older who fulfill the American Rheumatism Association preliminary classification criteria for systemic sclerosis. Must have at least moderate skin thickening (score of at least 2 by mRTSS) in at least one area suitable for repeat biopsy, such as arms, legs, or trunk	MEDI‐551 injections (0.1, 0.3, 1.0, 3, or 10 mg/kg) versus placebo	Completed, awaiting results; **Primary outcome measures** The safety and tolerability of MEDI‐551 will be assessed primarily by summarizing treatment‐emergent AEs and SAEs (at Day 85) **Secondary outcome measures** The secondary endpoints of the study are to assess the PK, IM, and PD of single IV doses of MEDI‐551 in adult subjects with scleroderma. Pharmacodynamics will be assessed by numbers of B cells in blood and skin (at day 85)	MEDI‐55 = anti‐CD19 monoclonal antibody
A study to evaluate safety and tolerability of multiple doses of MEDI‐546 in adult subjects with scleroderma (MEDI‐546) NCT00930683	Open‐label, single group assigned clinical trial, 15‐week	34 participants aged 18 and older who fulfill the American Rheumatism Association (ACR) preliminary classification criteria for Systemic Sclerosis with at least moderate skin thickening (score of at least 2 by mRTSS) in at least one area suitable for repeat biopsy, such as arms, legs or trunk.	MEDI‐546 (0.1, 0.3, 1.0, 3.0, or 10 mg/kg) injections	Completed, awaiting results; **Primary outcome measures** The safety and tolerability of MEDI‐546 will be assessed primarily by summarizing treatment‐emergent AEs and SAEs (time frame: study Day 84 for single‐dose; study Day 105 for multidose) **Secondary outcome measures** The secondary endpoints of the study are to assess the PK, IM, and PD of single and multiple IV doses of MEDI‐546 in adult subjects with scleroderma (time frame: study Day 84 for single‐dose; study Day 105 for multidose)	Human monoclonal antibody directed against type I interferon receptor
*Vasculature targets*					
Digital Ischemic Lesions in Scleroderma Treated With Oral Treprostinil Diethanolamine (DISTOL‐1) NCT00775463	Randomize, double blinded, placebo controlled, parallel assigned trial, phase 2, 20‐week	148 participants with SSc and presence of at least one active DU Treprostinil diethanolamine group (*n* = 71): • Age in years (range): 49.8 (19–82) • Sex: 54 females and 17 males • SSc Classification: 40 limited and 31 diffused • Disease duration in years (range): 10.3 (0–35) Placebo group (*n* = 76): • Age in years (range): 47.8 (20–74) • Sex:55 females and 21 males • SSc Classification: 55 limited and 21 diffused • Disease duration in years (range): 10.7 (0–30)	Treprostinil diethanolamine (0.25 mg titrated up to 16 mg) versus placebo, given orally twice daily for 20 weeks	Completed, with results; **Primary outcome measures** Treatment did not significantly changed the net ulcer burden (−1 (−1–0) vs. 0 (−1–1), *p* = .2) **Secondary outcome measures** Treatment significantly reduced the physician global assessment of DU severity (−26.7 (−43.3 to −5.3) vs. −13.3 (−31.3 to −1.3), ***p*** = **0.04)** No statistically significant difference between the two treatments in terms of: • mRSS (*p* = .68) • DU pain (*p* = .3) • Short‐Form McGill Pain Questionnaire (*p* = .54) • Patient global assessment of DU severity (*p* = .1) • Cochin Hand Function Scale at 20 weeks (*p* = .47) significance not measured for: • Scleroderma Health Assessment Questionnaire (SHAQ) • Patient Impression of Chance (PIC) Questionnaire • Short form 36 at Week 20 • Time to ulcer healing‐percentage of subjects with complete healing • AEs	Synthetic analogue of prostacyclin
Efficacy and safety of riociguat in patients with systemic sclerosis NCT02283762	Randomized, double blind, placebo controlled, parallel assigned trial, phase 2, 24‐month	117 participants with a mean age of 50.7 (12.2 *SD*). The female to male ratio was 3.17 (76% female). The average mRSS was 16.8 (3.7 SD).	Riociguat (0.5 mg titrated up to 2.5 mg over 10 weeks) vs placebo, given orally three times daily for 52 weeks Group 1: Riociguat—placebo Group 2: Placebo—riociguat	Completed, with results; **Primary outcome measured** Treatment did not significantly changed the mRSS at week 52 (−2.08 (5.65) vs. −0.77 (8.24), *p* = .08) **Secondary outcomes measured** Treatment significantly reduced the Physician's Global Assessment Score at Week 52 (−0.06 (2.15) vs. −0.74 (2.09), ***p*** = **0.02)** No significant difference between the two groups in terms of: • CRISS (ACR Composite Response Index for Clinical Trials) at Week 52 (*p* = .98) • Change in Health Assessment Questionnaire Disability Index (HAQ‐DI) Score at Week 52 (*p* = .35) • Change in Patient's Global Assessment Score at Week 52 (*p* = .08) • Change in FVC percent predicted at week 52 (*p* = .9)	Stimulation of soluble guanylate cyclase
Riociguat in Scleroderma Associated Digital Ulcers (RESCUE) NCT02915835	Randomized, quadruple blind, placebo controlled, parallel assigned trial, phase 2, 16 week	17 participants with a mean age of 51 years (18 *SD*). The female to male ratio of 13:4 (76.5% female). 9 participants had limited cutaneous (52.9%) and 8 participants had diffuse cutaneous (47.1%) The average time since first non‐Raynaud's symptom was 11.98 years (10.06 *SD*)	Riociguat (0.5 mg, 1 mg, 1.5 mg, 2 mg and 2.5 mg) versus placebo, given orally three times per day for 16 weeks (8 weeks titrating and 8 weeks stable dosing period)	Completed with results; **Primary outcome measures** Treatment did not significantly change the DU net burden (−1.22 (0.458) vs. −0.98 (0.425), *p* = .7) **Secondary outcome measures** Treatment did not significantly change the proportion of participants with/: • Healing of Cardinal DU (*p* = .61) • Healing of all DUs (*p* = 1.0) • No DUs at Week 16 (*p* = 1.0) • New active and indeterminate DU(s) over the study (*p* = 1.0) • Develop pressure ulcers (*p* = 1) • Healing of all pressure ulcers • Time to healing of cardinal or (*p* = .56) baseline DU (*p* = .35) • Raynaud's Condition Score (*p* = .76) • Raynaud's attacks/day (*p* = .57) • Duration of Raynaud's attacks (*p* = .4) • Patient's assessment of pain (*p* = .49), numbness (*p* = .75), tingling (*p* = .41) • During a Raynaud's attack • Physician's assessment of severity of Raynaud's disease (*p* = .31) or DUs (*p* = .84) • Patient's assessment of severity of Raynaud's disease (*p* = .11) or DUs (*p* = .66) or overall disease (*p* = .27) • Physician's global assessment for overall disease (*p* = .54) • PROMIS‐29 score • HAQ‐DI Score (*p* = .84)	Stimulator of soluble guanylate cyclase
Effect of sildenafil on the microcirculatory blood flow and endothelial progenitor cells in systemic sclerosis NCT01347008	Randomized, quadruple blinded, placebo controlled trial, phase 3, 8‐week	41 participants (21 in Sildenafil group, 20 in placebo group) with mean age of 44.5 years, 100% female population with Systemic sclerosis according to the ACR (1980) guidelines who have 6 or more Raynaud's crises per week	Sildenafil citrate 50 mg twice daily given orally for 8 weeks versus Placebo	Completed, awaiting results; **Primary outcome measured** Digital skin microvascular blood flow measured by LDI before cold stimulus Sildenafil group: 260.0 (108.0) Placebo: 246.3 (122.6) Digital skin microvascular blood flow measured by LDI after cold stimulus. Sildenafil group: 257.7 (123.3) Placebo: 220.5 (119.9) **Secondary outcomes measured** Daily frequency of Raynaud's Phenomenon attacks sildenafil group: 1.9 (2.4) Placebo: 1.7 (2.2)	PDE5 inhibitor
Sildenafil effect on digital ulcer healing in scleroderma SEDUCE Study (SEDUCE) NCT01295736	Placebo controlled, quadruple blinded RCT, 13‐week	84 participants age 18 and older with systemic sclerosis (ScS) according to classification criteria of ACR or of “LeRoy” and “Medsger” with at least one ongoing ischemic DU at baseline	Sildenafil 20 mg three times daily orally versus placebo	Completed, awaiting results; **Primary outcome measures** Time to healing of ischemic DUs **Secondary outcome measures** Time to healing of ischemic DUs at 90 days Change in number of ischemic DUs between baseline and day 90 Proportion of patients with complete healing of all DUs present at baseline and Day 90 Proportion of patients who do not develop any new DU after 28 days of treatment up to Day 90 Change between baseline and Day 90 in hand function and pain Proportion of patients with complicated DUs Evolution of the severity of Raynaud's phenomenon between baseline and Day 90	PDE5 inhibitor
A randomized control trial to assess the efficacy of tadalafil in Raynaud's phenomenon in scleroderma NCT01117298	Double‐blinded, placebo controlled clinical trial, 8‐week	66 participants between age 18 and 65 who have Raynaud's phenomenon secondary to scleroderma despite being on vasodilator therapy and presence of at least 3 of the 5 features of CREST syndrome.	Tadalafil 20 mg every other day for 8 weeks versus placebo	Completed, awaiting results; **Primary outcome measures** Daily frequency, duration and severity of Raynaud's phenomenon at baseline and at 8 weeks Appearance or healing of DUs at baseline and 8 weeks **Secondary outcome measures (at baseline and at 8 weeks)** Improvement in health assessment questionnaire Improvement in scleroderma specific health assessment questionnaire Improvement in quality of life Improvement in biomarkers of endothelial dysfunction Improvement in flow mediated dilatation	PDE5 inhibitor
Efficacy of botulinum toxin in scleroderma‐associated Raynaud's syndrome NCT02165111	Placebo controlled, quadruple blinded RCT, 4‐month	40 participants (one hand of each patient randomly selected for Botulinum A, other hand received placebo; total of 80 injections) with a mean age of 51.9 years and a female:male ratio of 3.44 who have diagnosis of scleroderma and have symptoms of Raynaud's syndrome affecting both hands.	Botulinum toxin A 20 units/ml injection versus Placebo (sterile saline) injection	Completed, awaiting results; **Primary outcome measured** Change in digital blood flow Botulinum group: −36.19 (−78.49–6.12) Placebo group: −6.10 (−46.28–34.07)) **Secondary outcomes measured** Change in Raynaud's phenomenon symptoms measured with the (Raynaud's Condition Score) Botulinum group: −0.18 (−0.22 to −0.13) Placebo group: ‐0.14 (−0.18 to −0.11) Number of ulcers Botulinum group: 0.45 (0.90), Placebo: 0.53 (0.93) Raynaud's symptoms severity Quick‐DASH Score Botulinum group: 26.96 (20.68) Placebo group: 29.11 (23.80) VAS for Pain Botulinum group: 2.68 (2.57) Placebo group: 3.05 (2.77) McCabe Cold Sensitivity Score Botulinum group: 185.31 (74.19) Placebo group: 86.76	Binds to presynaptic cholinergic nerve terminals and decreases release of acetylcholine
Diltiazem gel versus nitroglycerin ointment in healing process of scleroderma digital ulcers NCT02801305	Placebo controlled, double blinded, parallel assignedRCT, 6‐month	90 participants between age 20 and 70 who meet LeRoy criteria for scleroderma with at least one active DU	30 participants receive Diltiazem 2% ointment twice per day topically to ulcers for 8 weeks 30 participants receive nitroglycerin ointment 2% twice per day topically to ulcers for 8 weeks versus placebo (vaseline)	Completed, awaiting results; **Primary outcome measures** Assess effect of topical diltiazem on scleroderma DUs healing process **Secondary outcome measures** Assess significant difference in mean diameters of scleroderma DUs at 6 months Assess whether patients receiving diltiazem develop less new DUs over 6 months To assess difference in mean diameters of scleroderma DUs between placebo and nitroglycerin groups over 6 months To assess whether patients receiving nitroglycerin develop significantly less ulcers over 6 months To assess if patients receiving diltiazem have a significant difference in mean diameters of DUs versus nitroglycerin group To compare mean diameter of DUs according to their site in each group	Blocks voltage‐sensitive calcium channels in the blood vessels, by inhibiting the ion‐control gating mechanisms
Oral ifetroban to treat diffuse cutaneous systemic sclerosis (SSc) or SSc‐associated pulmonary arterial hypertension NCT02682511	Double blinded, placebo controlled RCT, 12‐month	34 participants age 18 to 80 with systemic sclerosis as defined using the 2013 ACR classification criteria and dcSSc within 7 years following initial diagnosis as defined by onset of the first non‐Raynaud's symptom.	Patients randomized to receive oral Ifetroban tablet or oral placebo daily for 365 days	Actively recruiting; **Primary outcome measures** Incidence of AEs and SAEs over 56 weeks **Secondary outcome measures** Change from baseline in FVC at 12, 26 and 52 weeks Change from baseline in diffusion capacity for carbon monoxide (DLCO) at 12, 26, and 52 weeks Change from baseline in the mRSS at 12, 26, 39, and 52 weeks Change from baseline in ventricular function as determined by cardiac MRI and echocardiography at 26 and 52 weeks Improve skin and peripheral vascular disease as measured by active DU count at 12, 26, 39, and 52 weeks	Thromboxane (TxA2) and prostaglandin H2 (PGH2) (TP) receptor antagonist
Placebo controlled trial of bosentan in scleroderma patients NCT00377455	Parallel assigned, double blinded placebo‐controlled trial, 12‐week	5 participants between 18 and 65 years. Female:male ratio of 5:0 (100% female) with diagnosis of SSc and >18 of the NYHA functional class I/II symptoms	Bosentan 62.5 mg BID for 1 month followed by 125 mg BID orally versus placebo	*Study terminated due to inadequate enrollment*	Competitive antagonist of endothelin −1
*Miscellaneous mechanism*					
Oral type I collagen for relieving scleroderma NCT00005675	Double blinded, parallel assigned RCT, 15‐month	168 participants aged 18 years and older with clinical diagnosis of diffuse systemic scleroderma (by ACR criteria 1980) of 10 years or less	500 mcg orally of type 1 collagen daily for 15 months versus placebo	Completed, awaiting results; **Primary outcome measures** Disease condition, as measured by physical exam, mRSS, health assessment questionnaires, chest X‐ray, and pulmonary function tests (time frame: measured at month 12)	Supports positive influence on chondrocyte function and works to create immune tolerance^168^
Scleroderma Treatment With Celution Processed Adipose Derived Regenerative Cells (STAR) NCT02396238	Double blinded, placebo controlled RCT, 48‐week	88 participants aged 18–70 years old with diagnosis of diffuse cutaneous scleroderma (duration >5 years) or limited cutaneous scleroderma Participants must have Cochin score ≥20 units and ability to safely undergo liposuction	ADRCs administered in two injections per digit on both hands versus placebo	Completed, awaiting results; **Primary outcome measures** Cochin score (time frame: 24 weeks) **Secondary outcome measures** Cochin score (time frame: 48 weeks) Raynaud's Condition Score (time frame: 24 weeks) Scleroderma Health Assessment Questionnaire (SHAQ) (time frame: 24 weeks) Physician and Patient Global Assessment (time frame: up to 48 weeks) Hand Mobility in Scleroderma (HAMIS) (time frame: up to 48 weeks) DU count (time frame: up to 48 weeks) Modified Rodnan Score (time frame: up to 48 weeks) Grip strength and pinch strength (time frame: up to 48 weeks) Finger circumference (with hand volume) (time frame: up to 48 weeks) 1st corner distance and sum of 2nd, 3rd, and 4th corner distances (time frame: up to 48 weeks) The EuroQOL five dimensions questionnaire (EQ‐5D) (time frame: up to 48 weeks) AEs, SAEs (time frame: up to 48 weeks)	ADRCs counteract inflammation, stimulate new blood vessel formation, prevent cell death, and secrete substances needed for repair and regeneration, all to reduce hand dysfunction
Cyclophosphamide and rATG With Hematopoietic Stem Cell Support in Systemic Scleroderma NCT00278525	Randomized phase II, parallel assigned CT, 12‐month	19 participants 60 years of age or less with established diagnosis of scleroderma, diffuse cutaneous scleroderma with involvement proximal to the elbow or knee and Rodnan score >14, plus any one of the following: DLCO <80% of predicted or decrease in lung function of 10% or more over past 12 months, active alveolitis on bronchoalveolar lavage, pulmonary fibrosis or alveolitis on CT scan or CXR, Renal disease not better explained by bacterial infection or other disorder, abnormal EKG or pericardial effusion or pericardial enhancement on MRI, gastrointestinal tract involvement confirmed on radiological study	Experimental group: stem cell transplantation (after conditioning regimen) versus standard of care	Completed, with results; **Primary outcome measures** Time to treatment failure (over 12 months) Definition: • Failure of skin score to improve (if >14 on enrollment) by a 25% above lowest post treatment value • Deterioration in diffusing capacity of DLCO, DLCO/alveolar volume (VA), or FVC by 10% below enrollment level or 10% below best post treatment value • Renal failure due to systemic sclerosis as defined by chronic dialysis ≥12 months • GI failure to to SSc as defined by initiation of TPN ≥12 months **Secondary outcome measures** Disease improvement (over 12 months) Definition: • At least 25% improvement in Rodnan skin score, or 10% improvement in PFT (DLCO, DLCO/VA, or FVC), or in cardiac tests (pulmonary artery (PA) systolic pressure by right heart cath) that persists >6 months or ability to wean off TPN	Cyclophosphamide = DNA alkylating agent rATG = antithymocyte globulin
Copper Impact on Venous Insufficiency and Lipodermatosclerosis (CIVIL) NCT03283800	Randomized parallel assigned double blinded CT, 8‐week	16 participants of any age with CEAP classification 4 in both legs and venous disease confirmed by venous duplex.	Copper impregnated compression stocking containing 2%–3% copper ions to be worn on one leg, daily for 8 weeks versus placebo	Completed, awaiting results; **Primary outcome measures** Aberdeen Varicose Vein Questionnaire (AVVQ) (time frame: 2 weeks) Venous Clinical Severity Scoring (VCSS) (time frame: 24 h) **Secondary outcome measures** Lipodermatosclerosis surface area [time frame: 2, 4, and 8 weeks after wearing compression stockings)	
Treatment of refractory sever systemic scleroderma by injection of allogeneic mesenchymal stem cells (MSC) NCT02213705	Single group assignment, nonrandomized phase I–II CT, 2‐years	20 participants age 18–70 years of age with established diagnosis of systemic sclerosis according to criteria of ACR. Patients SSc must be of poor prognosis, involving life‐threatening visceral impairment (cardiac, pulmonary, or renal) and (a) contraindicating the use of or (b) resistant to " immunosuppressive therapy conventionally used in severe forms of the disease according to the European recommendations of EUSTAR	One‐time injection of allogeneic mesenchymal stem cells	Unknown recruitment status, awaiting results; **Primary outcome measures** All grade 3 or above toxicity based on the CTCAE—Cancer Therapy Evaluation Program (CTEP), observed during the 2 years of follow‐up. **Secondary outcome measures** Survival (during 2‐year follow‐up) Progression free survival (during 2‐year follow‐up) Rodnan score (during 2‐year follow‐up) Clinical response and efficiency in the scalability of SSc (measured at 3, 6, 9, and 12 months after the procedure)	Mouse studies suggest reduction of collagen content and inflammation

Abbreviations: ACR, American College of Rheumatology; ADRC, adipose derived regenerative cell; AE, adverse event; DLCO, diffusing capacity for carbon monoxide; DU, digital ulcer; EULAR, European League Against Rheumatism; FVC, forced vital capacity; ITT, intention‐to‐treat; LDI, laser doppler imaging; MMF, mycophenolate mofetil; mRSS, modified Rodnan skin score; mRTSS, modified Rodnan Total Skin Score; PFD, pirfenidone; SAE, serious adverse event; SC, subcutaneous; SSc, systemic sclerosis.

*Note:* A comprehensive table of all current interventional clinical trials for cutaneous Scleroderma listed on clinicaltrials.gov. The table is broken down based on the mechanism of intervention being used as well as the status of the trial (e.g., *completed, active, terminated*, etc.).

## 2 ANTIFIBROTIC DRUGS

**Figure 1:**
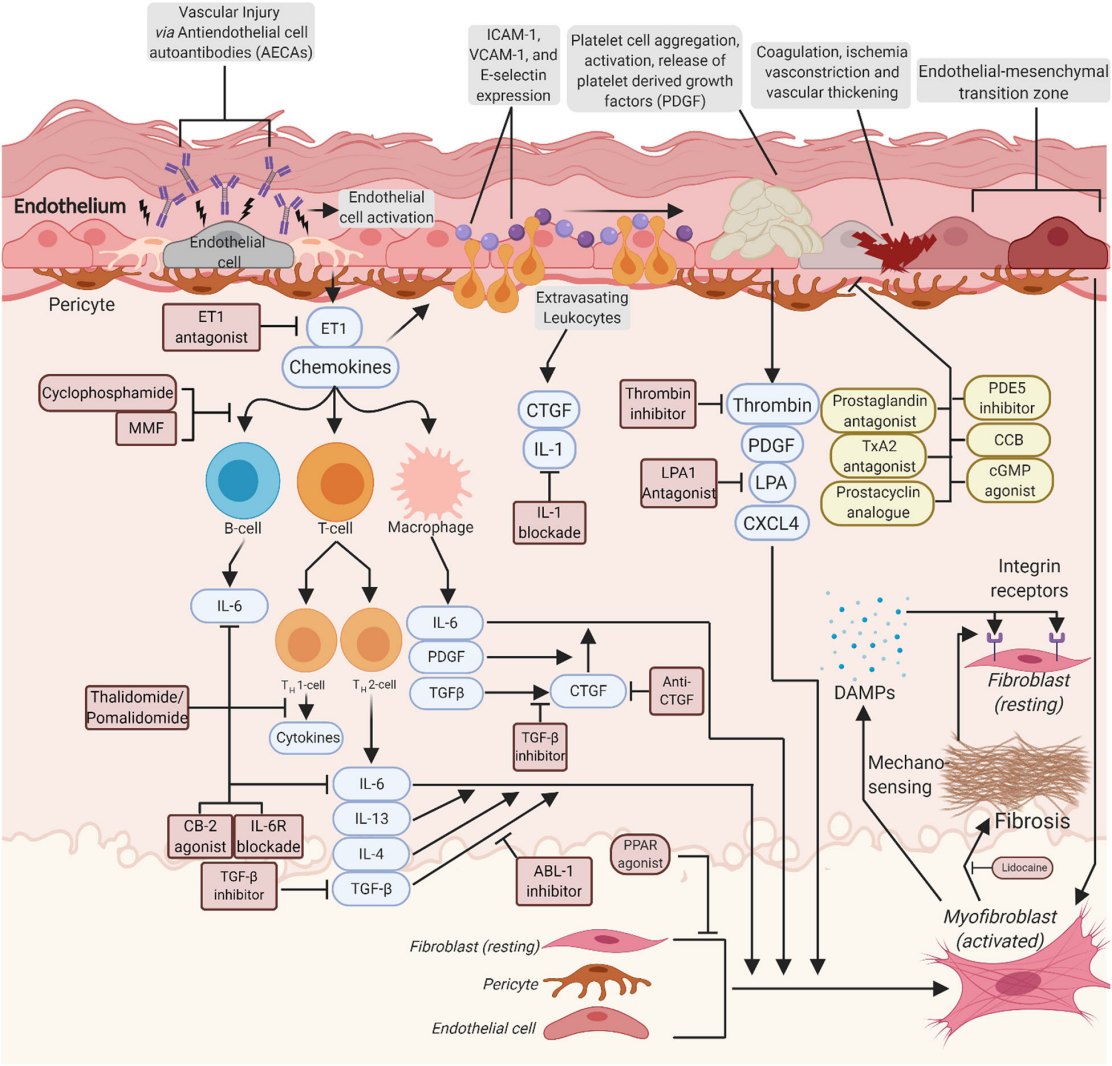
Potential drug targets for the treatment of morphea (created with Biorender.com). An unknown stimulus leading to tissue damage causes secretion of chemokines to recruit leukocytes to the dermis. Recruited leukocytes release cytokines including TGFβ and IL‐1, and IL‐6, in addition to IL‐4, 8, 13 (not shown), leading to differentiation of progenitor cells (fibroblasts, pericytes, and endothelial cells) into myofibroblasts. Abnormally activated myofibroblasts lead to excess production of collagen, fibronectin, and tenascin‐C, which act as DAMP and can activate undifferentiated fibroblasts into myofibroblasts. This self‐fibrotic process leads to uncontrolled proliferation of myofibroblasts. Here we have highlighted potential treatments for both morphea and scleroderma in yellow, and treatments used previously for scleroderma in pink. Antifibrotic targets for treatments include: TGFβ, tyrosine‐protein kinase, ABL‐1 inhibitor, anti‐CTGF, CB2 receptor agonist, PPAR‐γ and pan‐PPAR agonists, direct thrombin inhibitors. No current clinical trials for vascular targets exist for Morphea, but the 2017 EULAR treatment guidelines and current clinical trials for vascular agents targeting cutaneous manifestations of SSc are indicated. Anti‐inflammatory targets for treatments include: MMF, cyclophosphamide, CB2 receptor agonist, anti‐BAFF/BLyS (belimumab), anti‐CD20, thalidomide, IL‐6R blockade, abatacept, TGFβ inhibitor, PPAR agonist, UVA1 and UVB. CB2, cannabinoid‐2; CTGF, connective tissue growth factor; DAMP, damage associated molecular patterns; EULAR, European League Against Rheumatism; IL, interleukin; PPAR‐γ, peroxisome proliferator‐activated receptor γ; SSc, systemic sclerosis; TGFβ, transforming growth factor‐β

The histopathological hallmark of morphea is excessive deposition of extracellular matrix (ECM) in the skin, which is mainly composed of fibrillar collagens along with fibronectin, elastin, and tenascin C. This occurs as the result of inflammation‐triggered fibroproliferation and differentiation of fibroblasts into myofibroblasts (Figure 1).

Transforming growth factor‐β (TGFβ) is one of the most well‐studied profibrotic mediators in the skin.^12^ Fresolimumab, a humanized antibody targeting TGFβ, is well‐tolerated and had promising results in early stages of diffuse cutaneous SSc,^15^ making it a possible treatment for morphea. Intracellular TGFβ signaling involves both SMAD‐dependent and ‐independent pathways. The latter involves several tyrosine kinases, including ABL1, SRC kinase EGR1, signal transducer and activator of transcription 3 (STAT3), MRTFA, MRTFB, and FAK.^12^ Imatinib, an ABL1‐selective tyrosine kinase inhibitor, was not well‐tolerated and did not show satisfying anti‐fibrotic results in a clinical trial for treatment of SSc (Table 2). There is a trial of Imatinib ongoing for morphea patients (Table 1) with results pending.

Acting downstream of TGFβ, connective tissue growth factor (CTGF) works to stimulate fibrotic processes and is another potential therapeutic target for morphea.^16^ Elevated levels of CTGF messenger RNA (mRNA) has been reported in morphea patients.^17^ Iloprost, a prostacyclin analogue, has been found to temporarily suppress secretion of CTGF by fibroblasts.^16^ FG‐3013 (pamrevlumab), a fully recombinant human monoclonal antibody against CTGF was found to significantly attenuate peritoneal fibrosis in mouse models even below levels of fibrosis in CTGF knockout mice.^18^ FG‐3013 also inhibited recruitment of inflammatory cells, reduced vascular injury, and mitigated fibroblast migration. Numerous studies cite FG‐3013's ability to attenuate different models of induced fibrosis,^19^, ^20^ and a randomized controlled trial (RCT) of patients with interstitial lung fibrosis who were randomized to either FG‐3013 or placebo is awaiting trial results.^21^ These data warrant a clinical trial of pamrevlumab for morphea patients.

Lidocaine, is another intriguing treatment for scleroderma and is associated with an improvement in cutaneous fibrosis.^22^ Lidocaine reduces the activity of prolyl hydroxylase, an essential enzyme for the biosynthesis of collagen.^22^ It could be beneficial in treating morphea, especially considering topical delivery for localized disease.

Lipophosphatidic acid (LPA) is generated at sites of cell injury and induces various cellular responses including ECM production through LPA1 and LPA6 receptors.^23^, ^24^ SAR100842, a LPA1 receptor antagonist, reversed dermal thickening in a mouse model of skin fibrosis.^24^ A study of 15 patients with dcSSc who received SAR100842 showed a nonsignificant reduction in modified Rodnan skin score (mRSS), though the expression of LPA target genes were reduced in the active treatment group.^24^ Future studies of SAR100842 are warranted based on these results for SSc and morphea.

&Pirefenidone (PFD) is another agent that inhibits differentiation and proliferation of fibroblasts in animal models and in vitro studies.^25^, ^26^ A RCT is ongoing to examine PFD in SSc, and it could be of benefit for morphea patients.

## 3 ANTI‐INFLAMMATORY DRUGS

Both the innate and adaptive immune systems are involved in the inflammatory response in SSc as well as morphea. Various cytokines, chemokines, and inflammatory cell types are implicated in the cascade of events that lead to inflammation and release of profibrotic mediators. In the early stages of SSc and morphea, accumulation of monocytes, macrophages, T cells, B cells, mast cells, and eosinophils are observed histologically.^27^, ^28^ Here, we will breakdown different anti‐inflammatory targets for morphea based on their proposed mechanisms of action.

### 3.1 Agents targeting cytokines

IL‐6 and oncostatin M (OSM) are soluble mediators found in the serum of SSc patients and correlate with skin involvement.^12^ Both signal through the IL‐6 receptor (IL‐6R) heterodimer and utilize the Janus kinase (JAK)‐STAT pathway (see below).^29^ OSM mediates both fibrosis and inflammation via STAT3, which has been linked to endothelial cell activation and increased expression of adhesion molecules.^30^, ^31^ Targeting IL‐6 and its receptor had promising results in preclinical studies.^32^ Tocilizumab, an IL‐6 receptor antibody, showed modest effect on skin scores in SSc^12^ in a RCT. There are active clinical trials investigating the effect of another IL‐6R antibody, sarilumab, and GSK2330811, a humanized monoclonal antibody against OSM, on progression of morphea (NCT03679845) and dcSSc (NCT03041025), respectively.^33^

In a study of mRNA gene expressions from skin biopsies taken from patients with diffuse cutaneous SSc and localized SSc showed significantly elevated levels of IL‐7, IL‐13, and interferon γ (IFNγ),^34^ which seem to be potential targets. IL‐7 is a modulator of T and B cell development, with antifibrotic effects in SSc patients presumably through inhibition of TGFβ secretion from fibroblasts.^35^ IL‐13 has an important role in B cell function, monocyte modulation, and seemingly has the ability to suppress production of TNF, IL‐1, IL‐8, and chemokine (C‐C motif) ligand 3 (CCL3) from macrophages and monocytes.^36^, ^37^ Elevation of IL‐13 correlates with a profibrotic response, whereas IL‐7 and IFNγ worked to balance this response.^34^ IL‐1β, a member of the IL‐1 family, has associations with the pathogenesis of SSc.^38^ However, a recently completed RCT of rilonacept (IL‐1β inhibitor) (Table 2) showed no significant impact on cutaneous manifestations. Clinical trials targeting the other cytokines have not yet been conducted.

Polydeoxyribonucleotide (PDRN) exerts anti‐inflammatory responses by reducing TNF‐α, IL‐6, and high‐mobility group box protein‐1 (HMGB1),^39^ which have been positively correlated with skin thickness in SSc.^40^ The intramuscular form of PDRN, PLACENTEX, is currently being evaluated in SSc and could therefore be translated to morphea (NCT03388255).^41^

### 3.2 Chemokine targets

The selectivity of chemokines has been well‐studied, and their ability to recruit specific leukocytes has been the target of many pharmaceutical interventions. Monocyte chemoattractant protein 1 (MCP1, or CCL2) is produced by numerous immune cells and acts as the predominant chemoattractant and activator of monocytes and T‐cells.^42^ Elevated expression of CCL2 mRNA has been found in SSc fibroblasts.^42^ CCL3 also participates in the recruitment of monocytes and T helper lymphocytes, upregulates the expression of adhesion molecules, and correlates with a profibrotic response.^34^, ^43^ Interferon‐inducible T cell (I‐TAC, or CXCL11), a chemoattractant, has been found to attenuate bleomycin‐induced mouse model lung fibrosis with systemic treatment by inhibiting vascular remodeling.^44^ Inhibition of CCL24, a chemokine that regulates inflammatory and fibrotic activities through its receptor, CCR3, was shown to decrease liver fibrosis in patients^45^ and decrease activation of fibroblasts in bleomycin‐induced mouse model fibrosis.^46^ Fractalkine (CX3CL1), a chemokine expressed on proinflammatory cytokine activated endothelial cells and interacts with the receptor, CX3CR1, has been found to be strongly expressed in TGFβ‐cultured skin fibroblasts.^47^

CXCL4 was proposed to be an SSc biomarker that associates with progressive fibrosis in SSc patients,^48^ and correlates strongly with worsening pulmonary and skin involvement.^49^ CXCL4 is mostly secreted by plasmacytoid dendritic cells (pDCs), suggesting pDCs play a central pathological role in organ fibrosis.^49^ CXCL4 is widely accepted as one of the most antiangiogenic chemokines, which we have previously discussed as an early event in SSc.^49^ CXCL4 inhibits IFNγ, while increasing production of IL‐13 and IL‐4.^50^ CXCL4 also promotes monocyte survival,^51^ enhances monocyte binding to endothelial cell walls,^52^ and, when used to differentiate monocytes in‐vitro, strongly induces a specific macrophage subtype that is commonly seen in atherosclerotic plaques.^53^ Kroef et al.^54^ demonstrated that CXCL4 drives the release of PDGF, in mice bleomycin‐induced fibrosis models. PDGF release led to TNF‐α‐induced cytokine release^55^ and strongly activated fibroblasts.^48^ As our understanding of the pathological timeline in cutaneous SSc and morphea grows, the CXCL4‐PDGF mechanism will likely be a valid target for future clinical trials in homogenous subsets of patients.

There are currently no clinical trials targeted at inhibiting one or more chemokines or their receptors to our knowledge. These downstream mechanisms may play important roles in control of fibrosis in the future.

### 3.3 Agents targeting immune‐related receptors

Nuclear receptors, such as peroxisome proliferator‐activated receptors (PPARs), are another target implicating in the fibrogenesis in scleroderma.^56^, ^57^, ^58^ PPARγ, has a role in numerous cellular functions including regulation of cell growth, innate immunity, and connective tissue biology^59^ as it was shown to be a negative regulator of fibrosis both in vitro and in vivo.^60^, ^61^, ^62^, ^63^, ^64^, ^65^ PPARα is another target expressed in skin^66^ which participates in skin homeostasis, repair, and morphogenesis. Stimulating PPARα attenuates fibrogenesis in different organs.^67^, ^68^ Fenofibrate, a PPARα agonist, as well as rosiglitazone and pioglitazone, both PPARγ agonists, have shown promising results in reducing inflammation and skin fibrosis. It must be noted these medications have brought about safety concerns, which are suspected to be due to their strong agonistic activity.^58^, ^69^, ^70^ A novel pan PPAR‐agonist with moderate activity, IVA337 (lanifibranor), is currently being studied in a proof‐of‐concept clinical trial. IVA337 was developed to mediate some of the safety concerns, as well as to synergistically reduce profibrotic responses (Table 2). In vitro and in vivo preclinical studies have shown that IVA337 prevents, as well as induces regression of, fibrotic damage of skin.^71^, ^72^ If IVA337 continues to show efficacy and safety in its proof‐of‐concept trial, morphea patients could benefit from pan‐PPAR agonist drugs.

Cannabinoids exert anti‐inflammatory and anti‐fibrotic properties.^73^ They activate G‐protein‐coupled cannabinoid 1 and 2 (CB‐1 and CB‐2) receptors. CB‐2 found in skin and circulating immune cells.^56^ The CB‐2 agonist ajulemic acid (synthetic) has been found to block IL‐6 release, and moderate the response of fibroblasts through the generation of prostaglandin J2 and lipoxin A4 at low doses and direct stimulation of PPARγ at higher doses (see below).^57^, ^58^ A phase II clinical trial of Lenabasum (ajulemic acid) showed efficacy in patients with SSc^69^; however, another more recent trial did not show significant efficacy from Lenabasum versus placebo with preliminary data (Table 2).

### 3.4 Innate immune system targets

Toll‐like receptors (TLRs) are transmembrane receptors expressed on both innate immune and stromal cells that are important for sensing foreign compounds known as pathogen‐associated molecular patterns (PAMPs) as well as endogenous danger‐associated molecular patterns (DAMPs).^74^, ^75^ DAMPs are released after cellular stress and initial vascular injury in SSc and morphea and are the source of the subsequent inflammatory cascade.^76^ There is likely a propagation of DAMPs throughout the continuation of the disease, as they are released during ECM remodeling. Innumerable PAMPs and DAMPs trigger different TLRs and many have been found in increased levels in SSc patient serum and skin biopsies including S100A7 and the Epstein‐Barr virus.^77^, ^78^, ^79^, ^80^, ^81^, ^82^

Abnormal TLR signaling has been observed in SSc,^83^, ^84^, ^85^ but the related data for morphea patients has not been generated. TLR2 contributes to myofibroblast activation and differentiation.^86^ TLR2 recognizes its ligands by forming a heterodimer with TLR1 or TLR6. Deletion of TLR2 in bleomycin‐induced lung fibrosis mouse model,^87^ or blocking its downstream pathway by ablation of the adaptor protein MyD88 in human and mouse pericytes, is associated with reduced inflammation and fibrosis.^88^ A TLR2 blocking antibody, OPN‐305, has been also found to be safe and tolerable and may be useful in SSc.^86^, ^89^ TLR3 has been successfully blocked with CNTO 3157, a monoclonal antibody that inhibits dsRNA‐induced activation of NF‐κB response pathways, though no studies have used it in fibrosis models.^90^ A small subset of SSc patient biopsies showed significantly elevated levels of TLR3 in fibrotic skin lesions.^91^ Endothelin‐1 (ET‐1), a profibrotic molecule, that is activated by TLR3 is associated with enhanced pulmonary fibrosis in preclinical and clinical studies as well as skin fibrosis in mice.^92^ ET‐1 levels are increased in skin of SSc patients.^93^ Although there is no data on ET1 expression in morphea, it seems to be an auspicious target for skin fibrosis.^78^ TLR4 induces fibrosis via TGFβ signaling,^94^ and blocking its activity reversed fibrosis in various preclinical disease models. Expression of TLR4 and its ligands (HMGB1, heat‐shock protein 90, tenascin C, and fibronectin) is also increased in the skin of SSc patients, highlighting the therapeutic benefit of blocking TLR4. TLR8 has been recently proposed as a future target for SSc.^95^, ^96^, ^97^ TLR9 has strong association with TGFβ pathway and fibroblast activation in rapidly progressing idiopathic pulmonary fibrosis, suggesting a role in fibrosis.^98^ TLR inhibition may have greater effects earlier in the disease course due to the prevention of fibroblast transition as well as TGFβ secretion. While systemic inhibition of TLRs carries an increased risk of infection, topical application, or cell‐specific targeting, may prove a safer alternative for morphea patients.

A potential innate cell target in morphea is the dendritic cell (DC). DCs colocalize with fibroblasts and modulate their function and activity while expressing TGFβ1.^99^, ^100^ DCs express abnormally high levels of IL‐10 in SSc pathology.^101^, ^102^ Preclinical trials are being conducted to target DCs. Abatacept binds the costimulatory molecule CD86 on DCs and may work in part by blocking APC interactions with T‐cells (see below section on T cells).^103^ Existing therapies that block DCs such as Anti‐BDCA‐2 antibodies targeting pDCs and nanobodies may have clinical significance in morphea.^104^

Macrophages are one of the most abundant cells histologically in morphea lesions. They release proinflammatory and profibrotic mediators including TGFβ, PDGF, and IL‐6.^73^, ^105^ The early phases of skin sclerosis involve infiltration of leukocytes and macrophages.^106^ In patients with lSSc, Higashi‐Kuwata et al.^107^ showed an increased number of cells expressing CD204, which is expressed by M2 macrophages. Cyclic adenosine monophosphate (cAMP) is a ubiquitous second messenger molecule that is associated with numerous physiological responses including inflammation.^108^ The activation of cAMP has been well studied and is mediated by PDE‐4, which has been found to be expressed exclusively within inflammatory cells.^109^ In a study by Maier et al.,^73^ PDE‐4 inhibition was found to reduce skin fibrosis in different mice models of skin fibrosis. Inhibition of PDE4 resulted in decreased leukocytic infiltration in skin and decreased differentiation of M2 macrophages and their profibrotic cytokines.^73^ A Phase 2, pilot study is ongoing to assess the safety and efficacy of a topical PDE4 inhibitor, crisaborole 2% ointment for adult patients with morphea (Table 1).

### 3.5 Adaptive immune system

#### 3.5.1 Selective T cell‐targeted therapies

CD3^+^ and CD4^+^ T lymphocytes are the most abundant inflammatory cellular infiltrate in morphea.^110^ The first signal in T cell activation is antigen‐specific and is introduced via the T cell receptor and peptide‐MHC molecules on the APC membrane.^111^ The second signal is antigen nonspecific and is achieved via costimulatory molecules such as transmembrane protein CD28.^112^, ^113^ T cells that are activated without costimulation are susceptible to anergy, deletion, and dysregulated immune tolerance.^114^ CTLA4 is homologous to CD28, and both interact with CD80/CD86 on APCs.^115^ Preclinical data show that abatacept, a soluble recombinant fusion protein fused to the extracellular domain of CTLA‐4 and CD80/86, reduces skin fibrosis and cutaneous monocytes, B‐ and T‐cells in mice and circulating fibrocytes in an in vitro assay.^116^, ^117^ In early dcSSc, abatacept failed to improve skin score, though it showed a trend toward significance as well as manageable AEs.^118^ In contrast, numerous published cases exist for the use of abatacept in patients with morphea and show promise for future potential large‐scale trials.^119^, ^120^, ^121^ A retrospective compilation of eight cases of juvenile morphea showed no progression of disease after 30 months treatment with abatacept, and some cases had improvements in pre‐existing lesions.^122^ Blocking inducible T cell costimulator (ICOS), another member of the CD28‐superfamily, has shown promising antifibrotic activity in preclinical models of skin fibrosis.^123^ ICOS^+^ T cells are study was terminated elevated in the skin of patients with SSc.^123^ Further research is needed to identify safety and efficacy of anti‐ICOS agents in humans.

Cyclophosphamide is a traditional immunosuppressive alkylating agent that cross‐links cellular DNA and interferes with cell division.^124^ It is a well‐tolerated and approved drug for treatment of progressive skin disease and lung fibrosis.^12^ A recent clinical trial (Table 2) of patients with diffuse cutaneous SSc showed a significant improvement in mRSS with high dose cyclophosphamide (50 mg/kg). Cyclophosphamide might be a favorable agent for severe cases of diffuse morphea.

Basiliximab, a chimeric monoclonal antibody targeting CD25 (IL‐2Rα)‐positive lymphocytes, significantly reduced skin thickness in SSc patients.^125^ CD25 is expressed on activated T and B‐lymphocytes and morphea patients have elevated serum interleukin‐2 receptor levels. Of note, regulatory T cells (Tregs) also express high levels of CD25 and a few studies reported decrease in their number and activity in morphea^126^; thus, it is not clear what the net outcome of targeting CD25 in the disease activity would be. There is accumulating evidence that suggests Tregs are important for maintenance of self‐tolerance and prevention of autoimmunity. Frantz et al.^127^ raises the question that the paradoxical increase in Tregs seen later in SSc compared with the low number seen early in the disease suggests an over‐compensatory mechanism. Inhibition of Tregs later in the disease may prevent pathologic transition into effector T cells (Th17 and Th2). The immunosuppressive agent mycophenolate mofetil (MMF) works by inhibiting de novo synthesis of purines and suppresses T and B‐lymphocyte proliferation.^128^ Promising results have been seen (Table 2) in an RCT comparing MMF to placebo (plus cyclophosphamide) in terms of skin involvement.

Thalidomide, an immune modulator that works via suppressing Th1 cells, showed positive changes in skin fibrosis and number of infiltrated dermal CD8+ T‐cells in SSc patients.^129^ Pomalidomide is 100 times more potent than thalidomide.^130^ A previously active pomalidomide study was terminated due to not meeting the primary endpoints (Table 2). Overall, pomalidomide is a powerful immunomodulatory agent with numerous mechanisms including TNFα inhibition, while its use would be limited to severe cases of morphea due to the severe side effects including significant birth defects and sensorimotor peripheral neuropathy.

#### 3.5.2 Selective B cell‐targeted therapies

B‐cells have been implicated in SSc pathogenesis.^131^ Significant correlations have been found between both memory B cell phenotypes and autoantibodies with SSc severity, the specific antibodies are discussed elsewhere.^132^ B lymphocyte stimulator (BlyS), a B cell stimulating cytokine, has been also correlated with worsening skin fibrosis in patients with morphea.^133^ However, whether B cells and autoantibodies also play a role in morphea pathogenesis is less clear. Belimumab, a BlyS inhibitor, is currently being investigated in a single center placebo controlled RCT in patients with SSc (Table 2). However, it failed to show a significant reduction in cutaneous outcomes in a previous pilot trial.^134^

Depletion of B cells with rituximab (RTX), an anti‐CD20 monoclonal antibody, improved skin manifestations of SSc.^135^, ^136^, ^137^ A 5‐year open‐label trial evaluating B cell depletion therapy with RTX in morphea patients (Table 1) is still awaiting results. B cell depletion therapy has been shown to be effective in mouse model skin thickening and shows a promising therapy for human trials.^138^

## 4 DUAL ANTI‐INFLAMMATION AND ANTIFIBROTIC ACTIVITY

Phototherapy is the standard treatment for morphea, and it inhibits both fibrosis and inflammation. Current clinical trials include UVA‐1 versus placebo, medium versus high dose UVA‐1, UVB, and fractional carbon dioxide laser versus UVA‐1 phototherapy (Table 1).

**Figure 2:**
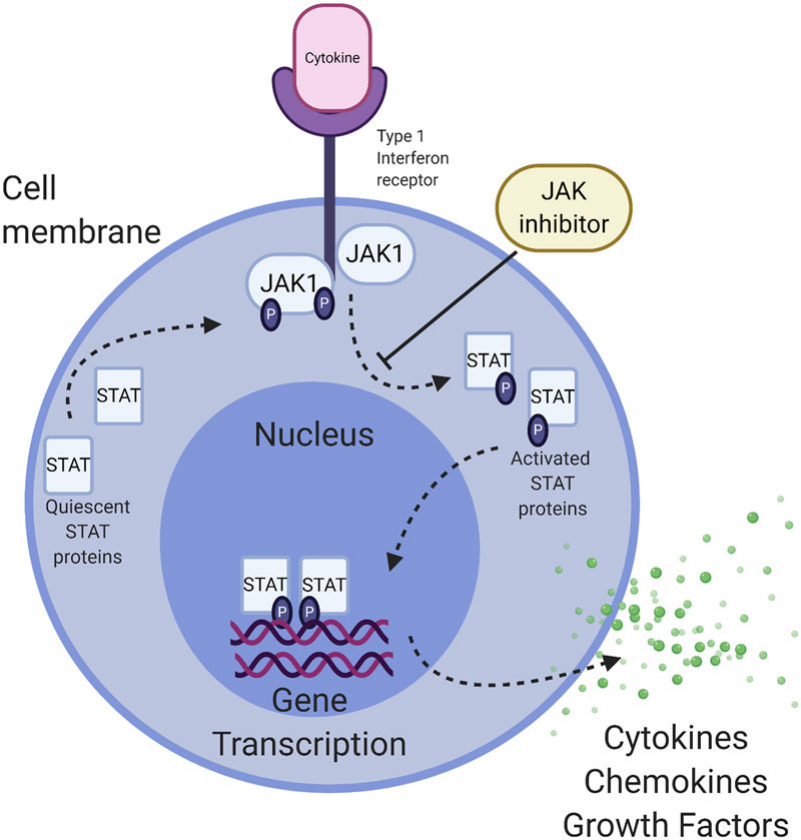
Targeting the JAK/STAT pathway for morphea (created with Biorender.com). Binding of cytokine to type‐1 interferon receptor activates downstream signaling pathways. Specifically, receptor dimerization causes subsequent phosphorylation of JAK and STAT proteins. STAT proteins (transcription factors) dimerize, translocate into the cell nucleus and regulate transcription of inflammatory genes such as IL‐2, IL‐4, IL‐6, IL‐12, IL‐21, IL‐22, IL‐23, IFN‐α, IFN‐β, and IFN‐γ, and oncostatin M. JAK inhibitors prevent JAK phosphorylation, STAT activation, and transcription of genes. GSK2330811, a novel oncostatin M inhibitor, has recently been tested in a double‐blinded, placebo controlled clinical trial in male and female participants with diffuse cutaneous SSc (dcSSc)

JAK‐STAT pathways play key roles in inflammation‐driven fibrosis (Figure 2). JAK/STAT pathways are activated by profibrotic cytokines and growth factors, IFNs,^139^ numerous interleukins^140^ and OSM (see above). STAT3 has a central role in TGFβ‐induced myofibroblast differentiation and collagen production, and selective STAT3 inhibitors are in development.^141^, ^142^ STAT4 deficient mice exhibited reduced fibrosis following bleomycin administration.^143^ Ruxolitinb, a JAK1 and JAK2 inhibitor, exerts anti‐fibrotic effects and was initially developed for myelofibrosis.^144^ Ruxolitinib has shown minimal responses in children with pan‐sclerotic morphea; however, one study found ruxolitinib was able to decrease scleroderma fibroblast activity downstream of TGFβ.^145^ In animal models, TGFβ‐induced fibrosis is JAK2 dependent.^145^, ^146^ JAK inhibition was seen to decrease inflammation in humans with morphea, some showing reversal of fibrosis as well.^147^ Baricitinib, another JAK1 and JAK2 inhibitor, had positive effects on skin fibrosis in one morphea patient^148^ while tofacitinib, a JAK1 and JAK3 inhibitor, showed substantial success in numerous morphea cases.^146^, ^149^, ^150^ A recently completed safety trial of tofacitinib showed a trend in improvement of skin fibrosis and no significant side effects over six months (Table 2). No JAK/STAT inhibitor has been examined at a large scale in morphea, though these case studies highlight their therapeutic potential.

## 5 CELLULAR AND GENE THERAPY

Cellular therapies including autologous hematopoietic stem cell transplantation have been effective in improving skin scores in selected SSc patients, and in a case report of two pediatric pansclerotic morphea patients.^70^, ^71^, ^72^, ^151^ Some clinical trials have begun to implement utilization of autologous cells in combination with gene editing therapy.

FCX‐013 is an autologous fibroblast, which is genetically modified using lentivirus and encoded for matrix metalloproteinase 1 (MMP‐1) that breaks down collagen. FCX‐013 is injected locally into fibrotic skin lesions, and the patient ingests an oral compound (Veledimex), which facilitates the MMP‐1 protein expression. A current trial has been fast‐tracked for the treatment of moderate to severe SSc (Table 2).

Gene editing technologies such as the clustered regularly interspaced sort palindromic repeats (CRISPR) system, have recently been used for manipulating the human genome and are effective for cutaneous diseases with a genetic component in preliminary research.^152^ Future advances may facilitate the use of CRISPR in morphea.

## 6 AGENTS TARGETING CELLULAR SENESCENCE, AGEING, AND DEATH: SENOLYTICS

Evidence suggests that premature activation of aging‐associated molecular mechanisms contribute to decreased self‐tolerance^153^ and the pathology of SSc^154^ and bleomycin‐induced models.^155^

Cellular senescence is a term used to describe a state in which there is irreversible growth arrest.^156^ A detectable phenotype for senescent cells, the senescence‐associated secretory phenotype (SASP), has been utilized in research to detect cellular senescence. In senescent fibroblasts, SASP has been associated with anti‐fibrotic effects in liver fibrosis mouse models.^157^ In a bleomycin‐induced lung fibrosis model, accumulation of senescent‐resistant myofibroblasts led to increased fibrosis.^158^

Dasatinib is a selective BCR‐Abl and Src family tyrosine kinase inhibitor. A recent analysis of a clinical trial of patients with SSc‐related‐interstitial lung disease showed decreased skin expression of SASP following dasatinib treatment correlated with clinical improvement.^159^ Additionally, patients with higher levels of SASP at baseline showed greater improvement after senolytic treatment,^160^ suggesting that clearance of pathogenic senescent cells in lesional tissue could be a measurable method of treatment response in future trials.

NADPH oxidase 4 (NOX4), a TGFβ responsive gene which is stimulated during the production of collagen, is another senolytic target.^12^ Activation of NOX4 leads to creation of reactive oxygen species, and expression of various genes required for collagen processing (*LOX, LOXL1‐LOXL4, P4Ha3, SERPINH1, FKBP10, PLOD2*), secretion, and ECM tissue deposition.^161^ GKT‐137831 (setanaxib), a NOX1/NOX4 inhibitor showed significant reduction in profibrotic TGFβ mediated effects on fibroblasts from sclerotic lesional tissue. Setanaxib is currently in a clinical trial for IPF patients,^162^ and shows promise as another senolytic pathway of potential utilization for morphea.

Mitochondrial priming,^163^, ^164^ a term used to describe the proximity of the cell's mitochondria to apoptotic activation, is driven by the detection of increased matrix stiffness in myofibroblasts, and is further mediated by the balance of pro‐ and antiapoptotic BCL‐2 family proteins.^165^ Increases in BCL‐2 family member expression leads to rapid elimination of myofibroblasts. This mechanism provides a model for resolution of tissue repair. In this model, cell survival becomes dependent entirely on BCL‐X_L_. It is hypothesized that this “stiffness activated” myofibroblast model is dependent on mechanotransduction (continued stimulation of myofibroblasts via ECM stiffness). ABT‐263 (navitoclax), a drug that displaces BCL‐X_L_ from proapoptotic BH3 homology BCL‐2 protein (BIM), successfully treats well‐established fibrosis in mouse models via induction of apoptosis.^166^ Bleomycin‐challenged mice have also been observed to have elevated levels of BCL‐2 versus controls. No clinical trials for navitoclax or other BH3 mimetics exist for SSc or morphea patients. A new therapeutic strategy of BH3 profiling to determine an individual's mitochondrial priming would allow researchers to stratify groups of patients.

## 7 MAJOR OPEN QUESTIONS

Questions remain in how morphea should be treated or managed, including: how can we safely test novel treatment approaches in clinical trials, if the goal is to limit inflammation early so as to prevent more extensive fibrosis? Testing novel therapeutics may require deviation from standard‐of‐care. Are there combination therapies or approaches that could prove better than standard of care? Ideally, halting inflammation and/or blocking immune‐fibroblast crosstalk would be combined with tissue remodeling to promote full homeostasis. How efficacious or translatable will senolytics be for morphea? Clinical trials specifically for morphea would need to be conducted.

## 8 CONCLUSIONS AND PERSPECTIVES

Though morphea can cause significant morbidity due to physical disfigurement and decreased quality of life, most clinical and translational research studies have focused on cutaneous manifestations of SSc. With recent advancements in our understanding inflammation, fibrosis, cellular senescence, advanced cellular aging, and dysregulated mechanisms of cellular control in morphea, we hope that successful morphea treatment research continues to look at ways in which we can halt and reverse inflammatory‐fibrotic processes. This will most likely be achieved through combination therapies that inhibit inflammation and reverse fibrosis. Ideal treatment regimens for morphea patients will ultimately improve safety and efficacy, potentially through topical/local administration.

## CONFLICTS OF INTERESTS

Jillian M. Richmond is an inventor on patent application #62489191, “Diagnosis and Treatment of Vitiligo” which covers targeting IL‐15 and Trm for the treatment of vitiligo, and on patent application #15/851,651, “Anti‐human CXCR3 antibodies for the Treatment of Vitiligo” which covers targeting CXCR3 for the treatment of vitiligo.

## AUTHOR CONTRIBUTIONS

*Roles: visualization; writing: original draft preparation; writing: review & editing*: Dan Wenzel. *Roles: writing—original draft preparation; writing—review & editing*: Nazgol‐Sadat Haddadi. *Roles: writing—review & editing*: Khashayar Afshari. *Roles: funding acquisition; writing—review & editing*: Jillian M. Richmond. *Roles: conceptualization, project administration; writing: review & editing*: Mehdi Rashighi.
